# Triggering receptor expressed on myeloid cells 2 deficiency exacerbates injury-induced inflammation in a mouse model of tauopathy

**DOI:** 10.3389/fimmu.2022.978423

**Published:** 2022-11-01

**Authors:** Atsuko Katsumoto, Olga N. Kokiko-Cochran, Shane M. Bemiller, Guixiang Xu, Richard M. Ransohoff, Bruce T. Lamb

**Affiliations:** ^1^ Department of Neurosciences, The Cleveland Clinic Lerner Research Institute, Cleveland, OH, United States; ^2^ Stark Neurosciences Research Institute, Indiana University School of Medicine, Indianapolis, IN, United States; ^3^ Department of Neurosciences, College of Medicine, The Ohio State University, Columbus, OH, United States; ^4^ Neuroinflammation Research Center, The Cleveland Clinic Lerner Research Institute, Cleveland, OH, United States

**Keywords:** TREM2, microglia, traumatic brain injury, tauopathy, neurodegeneration, bloodbrain barrier, blood-brain barrier

## Abstract

Traumatic brain injury (TBI) promotes several Alzheimer’s disease-like pathological features, including microtubule-associated protein tau (MAPT) accumulation within neurons. Macrophage activation in the injured hTau mouse model of tauopathy raises the question whether there is a relationship between MAPT pathology and alterations in macrophage activation following TBI. Triggering receptor expressed on myeloid cells 2 (TREM2) is a critical regulator of microglia and macrophage phenotype, but its mechanisms on TBI remain unclear. To address the association with TREM2 in TBI and MAPT pathology, we studied TREM2 deficiency in hTau mice (*hTau;Trem2^-/-^*) 3 (acute phase) and 120 (chronic phase) days after experimental TBI. At three days following injury, *hTau;Trem2^-/-^* mice exhibited reduced macrophage activation both in the cortex and hippocampus. However, to our surprise, *hTau;Trem2^-/-^* mice exposed to TBI augments macrophage accumulation in the corpus callosum and white matter near the site of tissue damage in a chronic phase, which results in exacerbated axonal injury, tau aggregation, and impaired neurogenesis. We further demonstrate that TREM2 deficiency in hTau injured mice promotes neuronal dystrophy in the white matter due to impaired phagocytosis of apoptotic cells. Remarkably, *hTau;Trem2^-/-^* exposed to TBI failed to restore blood-brain barrier integrity. These findings imply that TREM2 deficiency accelerates inflammation and neurodegeneration, accompanied by attenuated microglial phagocytosis and continuous blood-brain barrier (BBB) leakage, thus exacerbating tauopathy in hTau TBI mice.

## Introduction

Traumatic brain injury (TBI) triggers neuroinflammation involving brain resident microglia and peripherally derived macrophages. Cytokines, chemokines, and molecular mediators are released within hours of a TBI (1, 2), which leads to microglia and astrocytes’ activation and the recruitment of peripheral immune cells. These responses mainly promote the clearance of tissue debris and the resolution of the inflammatory response. However, it can cause secondary injury; a prolonged dysregulated microglial/macrophage activity results in neuronal death and chronic neurodegeneration, including oligomeric and hyperphosphorylated tau proteins (3–7) and amyloid beta oligomers (8, 9). Therefore, it is crucial to elucidate the mechanisms underlying detrimental myeloid cell inflammation and regulate these responses to prevent progressive neuronal damage.

Triggering receptor expressed on myeloid cells 2 (TREM2) is a type I transmembrane receptor found in dendritic cells, osteoclasts, microglia, monocytes, and tissue macrophages (10, 11). Homozygous mutations in either TREM2 or its adapter protein DNAX-activating protein of 12 kDa (DAP12) result in Nasu-Hakola disease (NHD), a progressive neurodegenerative disorder with massive gliosis and demyelination in the subcortical white matter (12, 13). Heterozygous rare variants in *TREM2* have been reported to increase the risk of Alzheimer’s disease (AD) (14, 15) and identified in other neurodegenerative disorders such as frontotemporal dementia and Parkinson’s disease as well (16, 17). Although exact mechanisms underlying NHD are unknown, it has been proposed that loss of TREM2 or DAP12 causes abnormalities in microglial survival, inflammatory response, and ability to clear neuronal debris (18, 19). TREM2 is also associated with hyperphosphorylated tau (20), another pathological hallmark for neurodegenerative diseases (21, 22). Silencing of brain TREM2 in the tau transgenic mice using a lentiviral-mediated strategy exacerbated tau pathology, possibly through neuroinflammation-induced hyperactivation of tau kinases (23). Using the same strategy, selective TREM2 overexpression on microglia was shown to ameliorate neuropathologies and spatial cognitive impairments in the tau transgenic mice, which suggests the protective role of TREM2 in tau pathology (24). However, the role of TREM2 in TBI is poorly understood. We recently showed that TBI increased TREM2 expression in C57BL/6 mice. After TBI, TREM2 deficient mice demonstrated reduced inflammatory cytokine production during the acute phase and reduced hippocampal atrophy during the chronic phase of recovery (25). TBI itself elicits tau phosphorylation (3, 5, 7). In addition, in a mouse model of tauopathy, the mice with TBI showed a persistent macrophage response and enhanced tau phosphorylation compared with controls (26, 27).

We focused on a dual outcome of TREM2 deficiency in the TBI and tau mouse model in the current study. We sought to determine the relative contribution of TREM2 in TBI-induced inflammation followed by tau accumulation. First, we evaluated the inflammatory response to TBI in *hTau;Trem2^-/-^* mice. Next, we assessed the progression of tau pathology and neurodegeneration in regions of the inflamed white matter in hTau;*Trem2* ^-/-^ TBI mice. Finally, we examined the blood-brain barrier integrity in hTau;*Trem2* ^-/-^ TBI mice to determine if the exacerbated tau pathology in a specific area is related to BBB impairment.

## Materials and methods

### Mouse

To study the effects of TBI-induced inflammation in promoting AD-like phenotypes, the human MAPT transgenic strain was mated to a separate *Mapt−/−* knockout mouse (Jackson Laboratory #007251) that was predicted to be a functional null allele (hTau mice). This mouse was maintained on the C57BL/6 background. We also used a *Trem2^−/−^* mouse model (Trem2tm1(KOMP)Vlcg) as previously described (28). These mice were maintained on a C57BL/6 background. These mice were crossed with hTau mice to yield hTau; *Trem2^LacZ/LacZ^* genotypes (also termed throughout the paper hTau; *Trem2^−/−^*). Two-month-old hTau mice and hTau; *Trem2^−/−^* mice (mixed sex; n = 6 mice per group for immunohistochemistry, n = 10-12 mice per group for behavior tests) were used for all studies. Animals were housed at the Cleveland Clinic Biological Resources Unit, and all procedures were approved by the Institutional Animal Care and Use Committee of the Cleveland Clinic.

### Surgical preparation and injury

We performed lateral fluid percussion injury to induce TBI as described before (25, 26, 29). Briefly, at two months of age, all mice were anesthetized with a combination of ketamine (100 mg/kg) and xylazine (10 mg/kg) and placed in a stereotaxic frame. Bupivacaine (0.25%, 50 μL) was administered subcutaneously before the midline incision. A 3.0 mm craniotomy was trephined over the right parietal cortex midway between bregma and lambda, leaving the dura intact. A modified Leur-Loc syringe (3.0 mm inside diameter) was placed over the exposed dura and held in place by cyanoacrylate adhesive. Twenty-four hours after surgical preparation, all mice were anesthetized with the same combination of ketamine and xylazine and connected to the fluid percussion injury (FPI) device. Animals in the injured group received a moderate level FPI (M = 1.0 atmospheres of extracranial pressure). Animals in the sham group were connected to the injury device; however, no fluid pulse was delivered. After FPI or sham injury, the syringe and adhesive were removed, and the incision was sutured. Mice were sacrificed at either 3 DPI or 120 DPI.

### Immunohistochemistry

Mice were deeply anesthetized with a combination of ketamine and xylazine and perfused with PBS. Brains were fixed overnight in 4% PFA in PBS, cryoprotected in 30% sucrose, and frozen in OCT. 30μm coronal sections were prepared on a cryostat and stored in PBS at 4°C. Matched medial and lateral sections were used for each animal. For 3,3′-diaminobenzidine (DAB) staining, endogenous peroxidases were quenched by incubating sections in 1% H_2_O_2_ in PBS for 30 min. Sections were blocked in 5% NGS/0.3% Triton X-100 in 1× PBS for 1 h. The following primary antibodies were added overnight at 4°C: Iba1 (1:1,000; Wako Pure Chemical Industries), CD45 (1:500; AbD Serotec), CD68(1:500; Bio-Rad), F4/80 (1:500; AbD Serotec), doublecortin (1:250; Cell Signaling), APP (1:1000; Invitrogen), and AT180 (1:500; Thermo Fisher Scientific). Mouse on Mouse Blocking Reagent (Vector Laboratories) was used at 1 µl/ml to block nonspecific staining with antibodies generated in mice and rats. Slices were incubated with appropriate biotinylated secondary antibodies (1:200; Vector Laboratories) and VECTASTAIN Elite ABC kit (Vector Laboratories) and developed with DAB with/without nickel chloride. For IgG staining, slices were incubated with biotinylated anti-mouse secondary antibody (1:200; Vector Laboratories) without blocking or primary antibody incubation. Slices were mounted with Permount (Thermo Fisher Scientific). For immunofluorescence, slices were permeabilized for 10min in PBS + 0.1% Triton X and antigen retrieval was performed at 85°C in 10mM sodium citrate pH6.0 for 15min. Slices were blocked in PBS with 5% NGS and 0.3% Triton X for 1 hour at room temperature. Slices were incubated with the following antibodies at 4°C overnight: Ubiquitin (1:2000, Thermo Fischer), Lamp1 (1:200, DSHB), AT8 (1:500; Thermo Fisher Scientific), Ki-67 (1:500, Abcam), Caspase-3 (1:500, Cell signaling), GFAP (1:500, Abcam). Slices were incubated for 1 hour at room temperature with Alexa flour conjugated secondary antibodies (1:1000) and coverslipped with Prolong Gold. For Fluoro-jade^®^ C (FJC)/DAPI, double staining was performed using Biosensis^®^ Fluoro-Jade C Ready-to-Dilute Staining Kit, following the manufacturer’s instructions. The final concentration of KMnO4 was 0.06% and 0.0001% for FJC. Gallyas-silver staining was performed as described (30). The slides were dehydrated again in graded ethanol for 2 min each. The slices were placed in xylene for another 5 min and coverslipped.

### Imaging and image quantification

Brightfield images were taken on a DMLS microscope (Leica), using a QImaging camera (QImaging) using LAX software (Leica). Fluorescent sections were imaged on a fluorescent Leica DM4000 microscope equipped with a QImaging Retiga EXi FAST 1394 MONO camera. For all experiments, 6 mice per group for each time point were analyzed. For measurement of area coverage, images of two random regions encompassing areas of interest per brain section and two sections at an interval of 300 μm per mouse were taken. Images were converted to grayscale after background subtraction in Adobe Photoshop to quantify sections. Adjusted grayscale images were then imported into Image J, and the appropriate threshold was applied to the image using the MaxEntrophy default threshold. The percentage of the area with immunoreactivity above the threshold was automatically calculated, and the same threshold cut-point was applied to images across the group. Because the differences between groups across the signal spectrum are not constant, information on the pixel intensity histograms for all experiments is shown in **Supplementary Figure 1**. Microglia phagocytosis was analyzed by assessing the co-localization of Iba-1 and Lamp-1 puncta. Using the ImageJ software Coloc2, Pearson’s correlation coefficient was calculated. For microglial morphology analysis, a total of 15–20 microglia were selected randomly from the white matter tract near the lesion from each group. Individual cells were thresholded, skeletonized, and analyzed for ramification (process length/cell and endpoints/cell) using the ImageJ AnalyzeSkeleton plugin, which outputs a total number of branches along with branch junctions. Fractal analysis (FracLac for ImageJ) was used to quantify cell complexity (fractal dimension), cell shape (span ratio), and cell size (density). All data acquisition and analysis were made in a blinded manner.

### Statistical analysis

All data are presented as mean ± standard error of the mean (SEM). All statistical analysis was completed with GraphPad Prism 5 software (Sandiego, CA). A mixed model factorial analysis of variance (ANOVA) followed by the Bonferroni *post hoc* test was used to evaluate group differences. Comparisons between two groups were analyzed using Student’s *t-test* (two-tailed; unpaired) at a 95% confidence interval. Statistical significance was determined at *p* < 0.05*, *P* < 0.01**, *p* < 0.005***, and *p* < 0.001****.

## Results

### TREM2 deficiency in hTau TBI mice attenuates macrophage activation in the acute phase (3 days post-injury)

**Figure 1:**
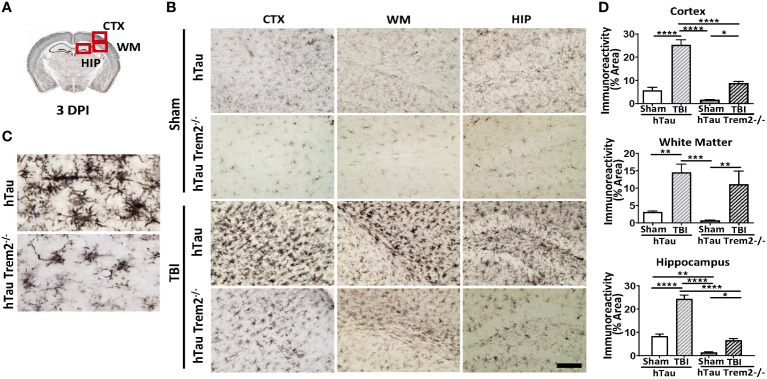
Traumatic brain injury (TBI) induces enhanced microglial/macrophage response in hTau mice and hTau;*Trem2^-/-^* mice at 3 days post-injury (DPI). **(A)** The diagram shows areas where images were taken from. CTX, cortex; HIP, hippocampus; WM, white matter. **(B)** Representative images of F4/80 immunostaining in sham and TBI of hTau and hTau;*Trem2^-/-^* mice in the lateral CTX, WM, and HIP at 3 DPI. **(C)** Magnified view of F4/80 immunostaining in hTau TBI mice and hTau;*Trem2^-/-^* TBI in the lateral CTX. **(D)** Quantification of percent area covered by F4/80. All experiments used n = 6 (equal males and females) mice per group unless otherwise noted. At least two independent experiments were performed for each analysis. The scale bar represents 50μm. Error bars indicate SEM; *p < 0.05, **p < 0.01, ***p < 0.005, ****p < 0.001.

To address the effect of *TREM2* deficiency on TBI-induced neuroinflammation in the mouse model of tauopathy, hTau and hTau;*Trem2^-/-^* mice were subjected to moderate lateral FPI, and the inflammatory reaction was analyzed three days post-injury (dpi) by immunohistochemistry (**Figure 1**). In hTau mice, macrophage and microglial marker F4/80 expression was massive in the injured site (**Figures 1B, D**). In addition, magnifying observation revealed the increased F4/80 expression in the cortex, the corpus callosum, and the hippocampus compared to sham-operated mice (**Figure 1C**). Similarly, hTau;*Trem2^-/-^* mice showed increased F4/80 expression in the injured brain. However, this myeloid cell activation was significantly reduced in the cortex and hippocampus of hTau;*Trem2^-/-^* TBI mice compared to hTau-TBI mice (**Figures 1B, D**).

### TREM2 loss of function in hTau TBI mice leads to sustained inflammation in the chronic phase (120 days post-injury)

**Figure 2:**
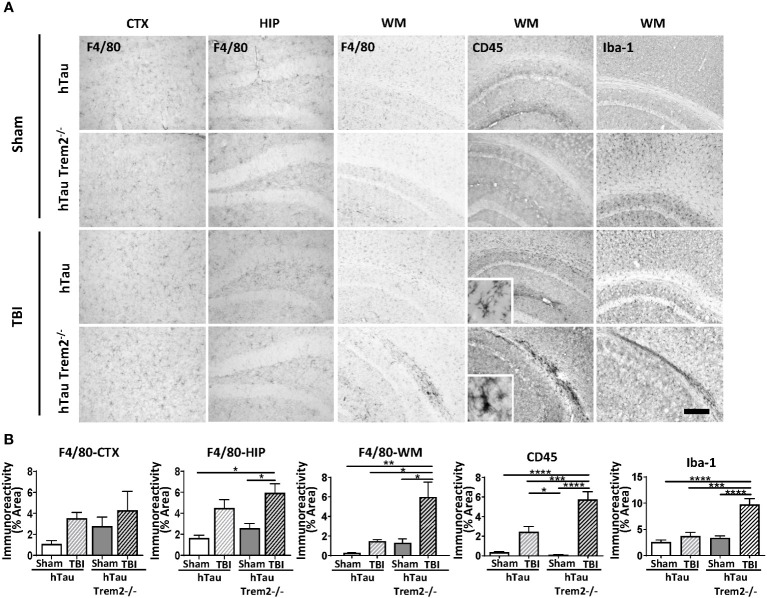
HTau;*Trem2^-/-^* TBI mice showed enhanced Iba1, CD45, and F4/80 in the white matter tract near the lesion at 120 DPI. **(A)** Representative images of CD45, F4/80, and Iba-1 immunostaining in sham and TBI of hTau and hTau;*Trem2^-/-^* mice in the cortex, hippocampus, and white matter tract near lesion site. **(B)** Quantification of percent area covered by F4/80, CD45, and Iba-1. TBI significantly increased this immunoreactivity in hTau;*Trem2^-/-^* TBI mice compared with all other groups in the lateral cortex. All experiments used n = 6 (equal males and females) mice per group unless otherwise noted. At least two independent experiments were performed for each analysis. Scale bar represents 100μm. Error bars indicate SEM; *p < 0.05, **p < 0.01, ***p < 0.005, ****p < 0.001.

The previous report showed that TREM2 has different roles in AD-related myeloid cell functions early and late in disease progression (31). Therefore, we next assessed whether TREM2 deficiency might differentially affect neuroinflammation in hTau mice at a later time point. At 120 dpi, which represents a chronic time point that is also consistent with our previous work (7, 25, 26, 32, 33), accumulation of cells positive for macrophage markers, F4/80, CD45, Iba-1, and CD68, were robust in the corpus callosum and white matter near the site of tissue damage in hTau;*Trem2^-/-^* -injured mice (**Figure 2A**; **Supplementary Figure 2A**). These immunoreactive areas were significantly increased in TREM2 deficient TBI mice (**Figure 2B**). This was accompanied by astrocytosis shown by GFAP immunostaining (**Supplementary Figure 2B**). Morphological analysis revealed that Iba-1+ cell ramification was decreased in hTau;*Trem2^−/−^* TBI mice compared to hTau;*Trem2^−/−^* sham mice, and there was a significant increase in the density both in hTau TBI mice and hTau;*Trem2^−/−^* TBI mice. (**Supplementary Figure 3**). Overall, we observed a consistent immunoreactive pattern for microglia/macrophages in hTau;*Trem2^-/-^* TBI mice.

### TREM2 deficiency in hTau mice promotes TBI-induced axonal damage and impairs neurogenesis in the chronic phase

**Figure 3:**
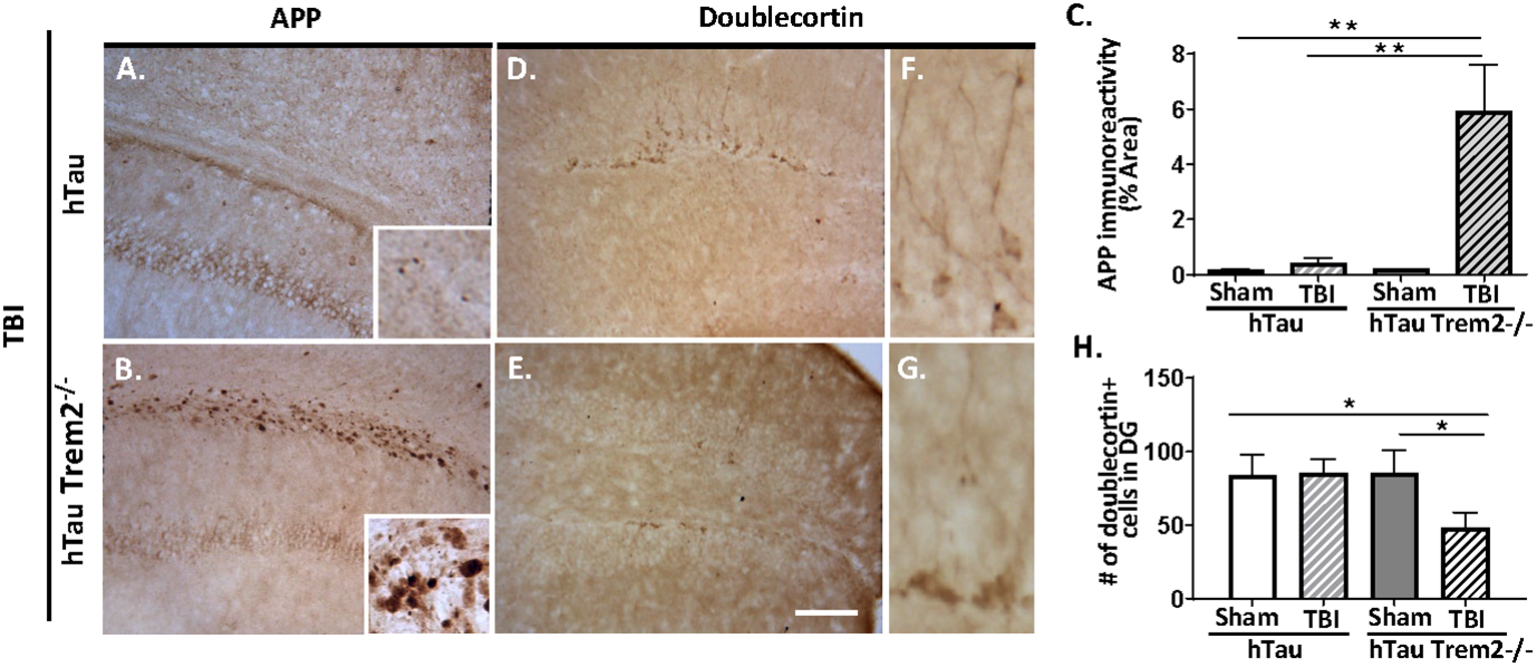
The axonal injury was accelerated in the white matter, and neurogenesis was impaired in injured hTau;*Trem2^-/-^* mice at 120 DPI. **(A, B)** Representative images of APP immunostaining in hTau TBI and hTau;*Trem2^-/-^* TBI mice in the external capsule near the lesion site. **(C)** Quantification of percent area covered by APP. **(D, E)** Representative images of doublecortin immunostaining in hTau TBI and hTau;*Trem2^-/-^* TBI mice in the hippocampus. **(F, G)** Magnified view of **(D, E)**. **(H)** Quantification of the number of doublecortin positive cells identified impaired neurogenesis in hTau;*Trem2^-/-^* TBI mice. All experiments used n = 6 (equal males and females) mice per group unless otherwise noted. At least two independent experiments were performed for each analysis. The scale bar represents 50μm. Error bars indicate SEM; *p < 0.05, **p < 0.01.

Axonal injury is an important pathological feature of TBI, and beta-amyloid precursor protein (APP) immunohistochemistry was performed to assess impaired axonal transport. Consistent with previous studies (7, 34), APP immunoreactivity was enhanced in the body of the corpus callosum and the external capsule in injured hTau mice (**Figure 3A**). Notably, hTau;*Trem2^-/-^* mice showed prominent APP staining in the corpus callosum and the external capsule linked with it compared to the injured hTau mice (**Figure 3B**). Higher magnification showed APP accumulation in axonal bulbs or swellings in this area. Quantifying axonal pathology as the ratio of APP immunoreactivity in areas including corpus callosum documented that axonal pathology was significantly worsened in hTau;*Trem2^-/-^* mice compared to hTau mice (**Figure 3C**). These observations indicate that TREM2 deficiency increases axonal injury, as monitored with APP immunoreactivity, after TBI in hTau mice.

Neurogenesis is also affected by TREM2 deficiency in hTau TBI mice. Immunohistochemistry and quantitative morphometry for neuroblast marker the neuronal migration protein, doublecortin (DCX), in hTau and hTau;*Trem2^-/-^* groups of mice showed a significant decrease in DCX in injured hTau;*Trem2^-/-^* mice, suggesting impaired neurogenesis (**Figures 3D, E and H**). Furthermore, the hTau;*Trem2*-/- mouse showed immature cells with short neurites, while the hTau mouse demonstrated more developed cells with branches (**Figures 3F, G**).

### Both injury and TREM2 genotype affect aggregated Tau levels in the chronic phase

**Figure 4:**
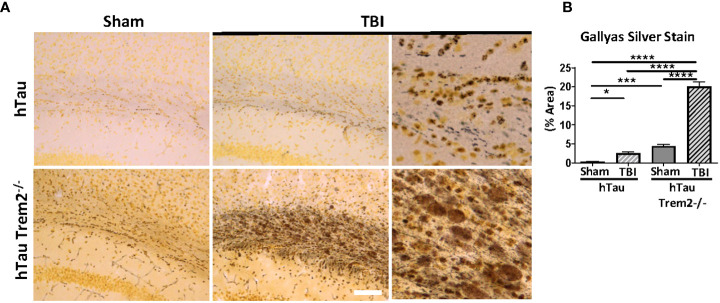
Prominent tau aggregation in the white matter near injury site in injured hTau;*Trem2^-/-^* mice in chronic phase. **(A)** Aggregated tau was identified using Gallyas silver staining. Images were acquired in the external capsule near the lesion site in hTau and hTau;*Trem2^-/-^* mice with or without TBI. **(B)** Silver staining was significantly enhanced in the white matter of hTau;*Trem2^-/-^* TBI mice compared with hTau TBI mice. All experiments used n = 6 (equal males and females) mice per group unless otherwise noted. At least two independent experiments were performed for each analysis. The scale bar represents 50μm. Error bars indicate SEM; *p < 0.05, ***p < 0.005, ****p < 0.001.

To determine whether hTau;*Trem2^-/-^* TBI mice display accumulation of aggregated tau, Gallyas silver staining was performed (**Figure 4**). In response to TBI, increased staining at the injury site in the white matter of both hTau and hTau;*Trem2^-/-^* mice (**Figure 4A**) were observed. Increased tau aggregation was also detected in hTau;*Trem2^-/-^* mice with sham. Strikingly, more prominent Gallyas positive neurons in white matter and dystrophic neurites were identified in hTau;*Trem2^-/-^* TBI mice (**Figures 4A, B**). Compared to small, granular staining in hTau TBI and hTau;*Trem2^-/-^* sham mice, hTau;*Trem2^-/-^* TBI mice showed larger and speckled staining. Because tau aggregates in the hTau mice appear after nine months of age (35), our data demonstrate that pathologic tau aggregation is affected in the hTau;*Trem2^-/-^* mice, and the injury further accelerates it.

### Injury accelerates neurodegeneration in hTau;Trem2^-/-^ mice in the chronic phase

**Figure 5:**
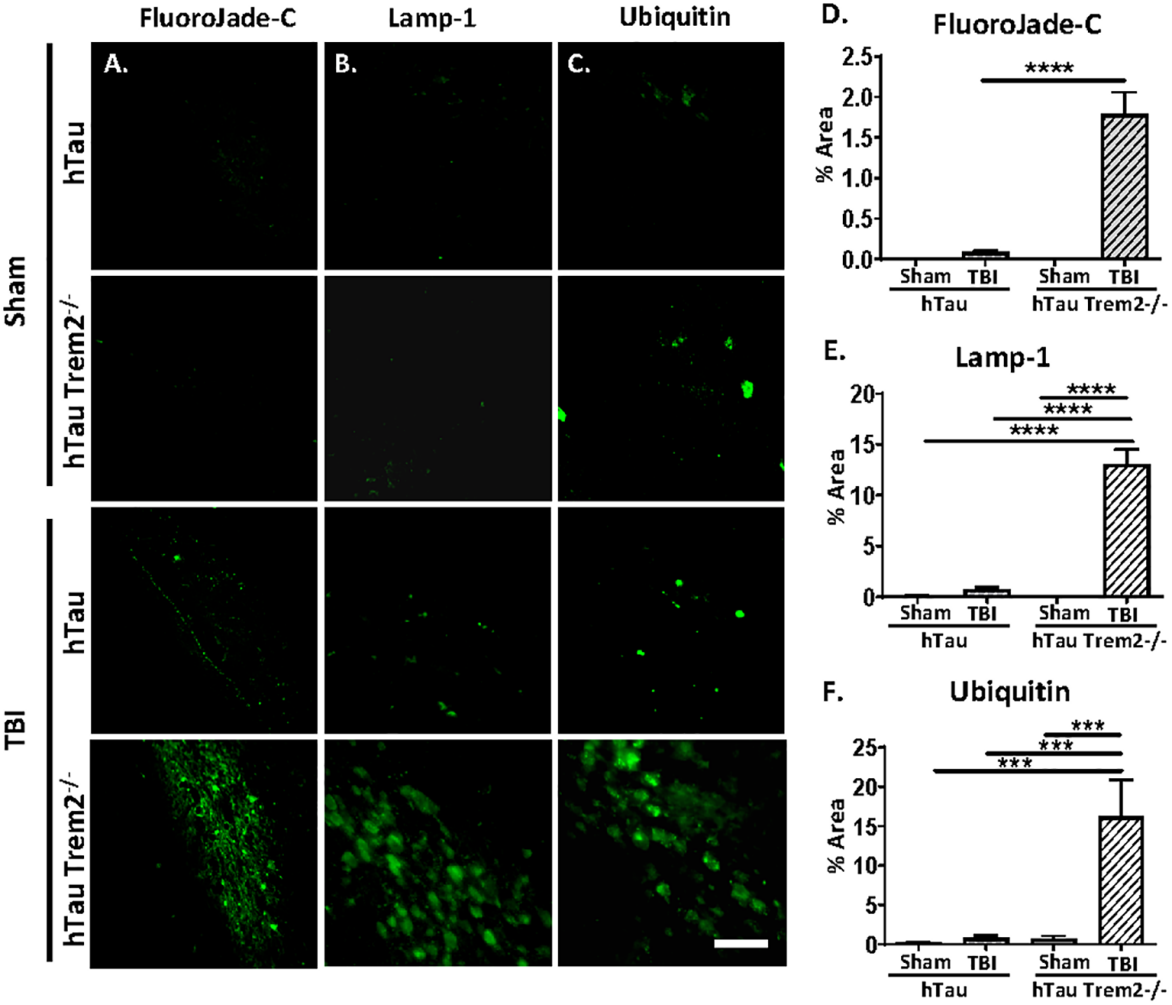
The swollen dystrophic neurons in the white matter in hTau;*Trem2^-/-^* TBI mice in the chronic phase. Representative images of FluoroJade-C (**column A**), Lamp-1 (**column B**), and ubiquitin (**column C**) from hTau and hTau*Trem2^-/-^* mice with or without TBI. **(D–F)** A significant increase in neurodegeneration was found in hTau;*Trem2^-/-^* TBI mice. ND. Not detected. All experiments used n = 6 (equal males and females) mice per group unless otherwise noted. At least two independent experiments were performed for each analysis. The scale bar represents 50μm. Error bars indicate SEM; ***p < 0.005, ****p < 0.001.

Next, we assessed whether the white matter’s inflammation and tau aggregation led to neurodegeneration by immunohistochemistry for the degenerating neuron-specific antigen fluoroJade-C (FJC) in hTau and hTau;*Trem2^-/-^* groups of mice (**Figure 5A**). Surprisingly, there were significant levels of degenerating neurons within the white matter near the site of tissue damage in the hTau;*Trem2^-/-^* TBI group compared to the hTau TBI group (**Figure 5A**). No FJC-positive cells were observed in sham groups. Quantifying the FJC^+^ area demonstrated a significant increase in the hTau;*Trem2^-/-^* TBI mice compared to hTau TBI mice (**Figure 5B**).

In addition, immunolabelling of dystrophic neurites using anti-Lysosome-associated membrane glycoprotein 1 (Lamp-1) or anti-Ubiquitin antibodies showed robust numbers of the swollen dystrophic neurites in the white matter near the site of tissue damage (**Figures 5C, D** and quantified in **5E, F**).

### Macrophage phagocytic function is impaired in hTau;Trem2^-/-^ injured mice in the chronic phase

**Figure 6:**
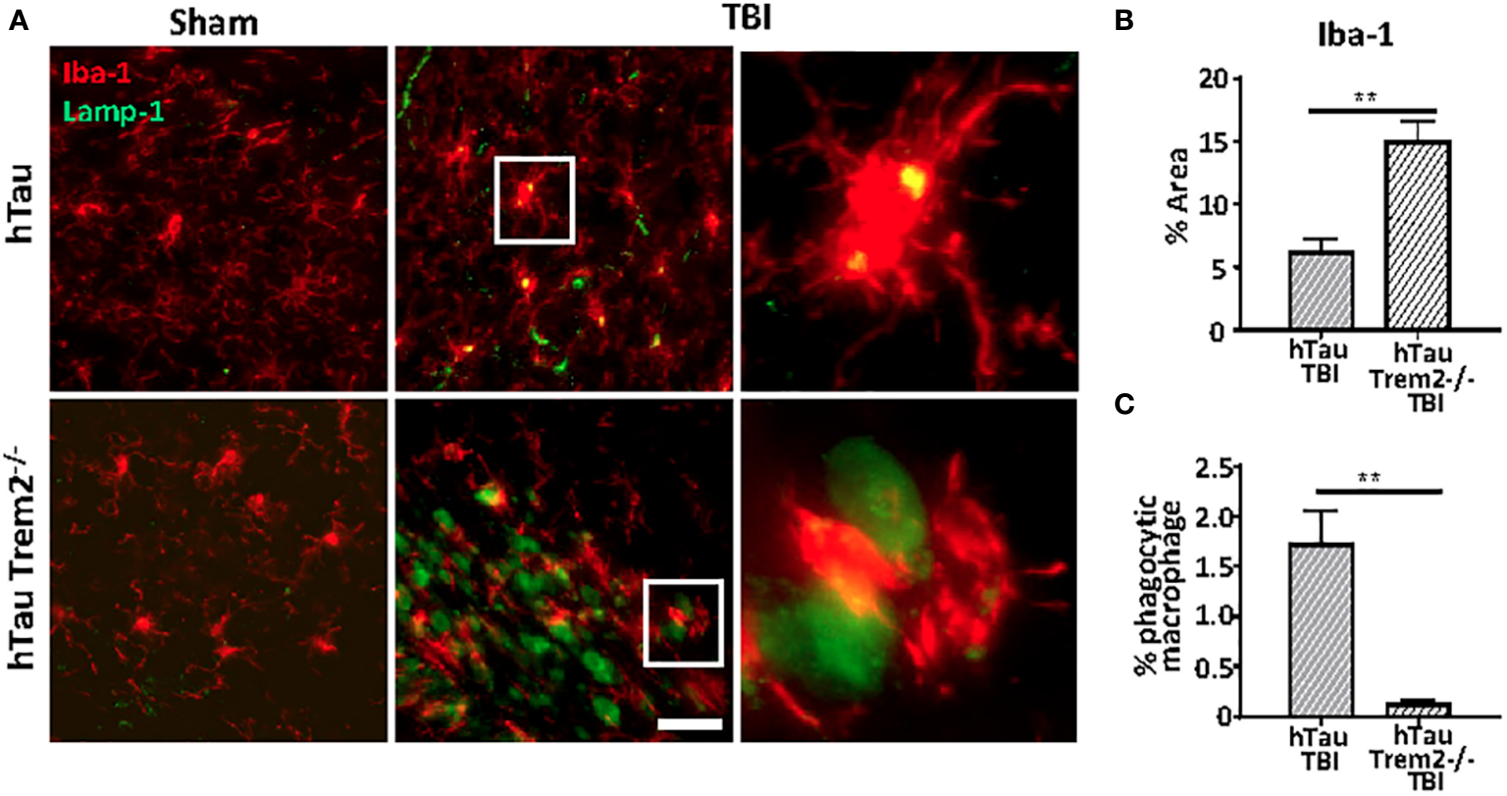
The phagocytic function was impaired in hTau;*Trem2^-/-^* TBI mice compared with hTau TBI mice in the chronic phase. **(A)** Representative images of Iba-1 (red) and Lamp-1 (green) in sham (far left column) and TBI (middle column). Enlarged images of the TBI group are in the far-right column. Although the number of Iba-1 positive microglia/macrophages was increased in hTau;*Trem2^-/-^* mice **(B)**, the percent of Iba-1^+^ cells that contained Lamp-1^+^ elements was significantly low in hTau;*Trem2^-/-^* TBI mice relative to hTau TBI controls **(C)**. All experiments used n = 6 (equal males and females) mice per group unless otherwise noted. At least two independent experiments were performed for each analysis. The scale bar represents 20μm. Error bars indicate SEM; **p < 0.01.

TREM2-positive myeloid cells are involved in tissue debris clearance (36–38) and amyloid clearance (19, 39). Therefore, to examine whether TREM2 deficiency could lead to compromised phagocytic capacity, Iba-1 positive cells co-localization with Lamp-1-positive dystrophic neurites in the white matter tract near lesion were analyzed (**Figure 6A**). Although Iba-1 positive cells were prominent (**Figure 6B**), Lamp1-positive phagocytic macrophages were significantly less in hTau;*Trem2*-/- injured mice (**Figure 6C**).

### Abnormal blood-brain barrier leakage in hTau;Trem2^-/-^ mice in chronic phase

**Figure 7:**
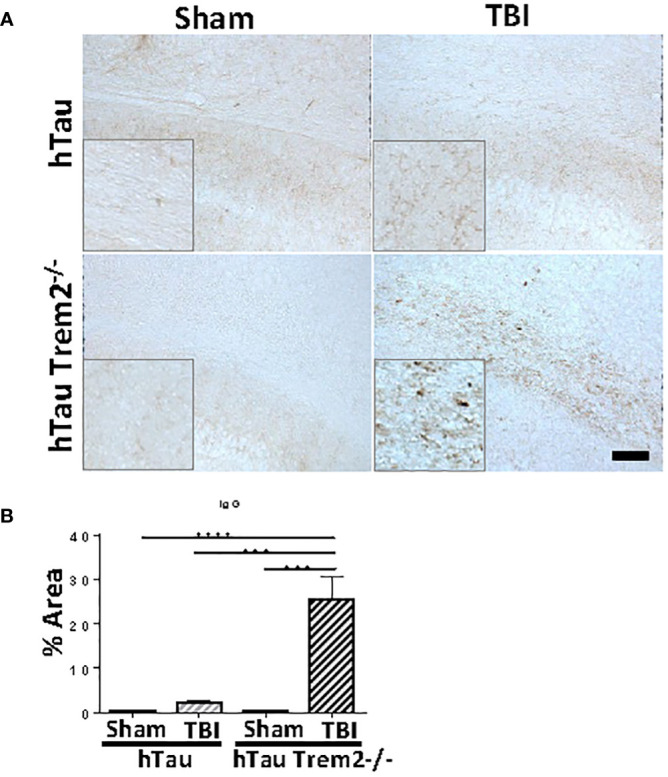
Significant increase of blood-brain barrier leakage in the white matter near the site of tissue damage in hTau;*Trem2^-/-^* mice, but not in other groups. **(A)** Representative images of hTau and hTau;*Trem2^-/-^* mice with or without TBI stained with IgG in the chronic phase. **(B)** Quantification of IgG staining. All experiments used n = 6 (equal males and females) mice per group unless otherwise noted. At least two independent experiments were performed for each analysis. The scale bar represents 100μm. Error bars indicate SEM; ***p < 0.005, ****p < 0.001.

Given our finding that TREM2 deficiency leads to chronic inflammation and neurodegeneration in a specific area, we hypothesized that this area would be sites of inflammatory cells and molecules entry. Apart from the normal staining in periventricular structures, IgG staining demonstrated a clear and significant increase of BBB leakage in the white matter near the site of tissue damage in hTau;*Trem2^-/-^* mice, but not in other groups (**Figure 7**).

## Discussion

In this study, we investigated hTau mice with a loss-of-function mutation in TREM2 both in the acute and chronic phases after TBI. TREM2 deficiency in hTau mice reduced cortical and hippocampal myeloid cell immunoreactivity near the injury site during the acute phase of TBI. During the chronic phase of recovery, reactive myeloid cells accumulate in the white matter tract of the corpus callosum, specifically deep in the injured region. This area in hTau;*Trem2^-/-^* TBI mice had severe neuronal damage and tau aggregation, accompanied by degenerating neurons and dystrophic neurites. Furthermore, microglia/macrophage in hTau;*Trem2^-/-^* mice failed to phagocytose degrading neurons in the white matter (**Table 1**).

**Table 1:** TREM2 functions in different genetic backgrounds.

		B6 TBI	Trem2^-/-^ TBI (25)	hTau TBI (24)	hTau;Trem2^-/-^ TBI ^[our study]^
Acute phase	Microglial/ macrophage activation	+	Less	More	Less
Chronic phase		_	_	+	Robust in white matter
	Phagocytic function	N/A	N/A	Preserved	Impaired
	BBB restoration	N/A	N/A	Proper	Impaired
	Brain atrophy	+	Less	Not significant	
	Tauopathy	_	N/A	+	Accelerated
	Cognition	Impaired	Rescued	Compromised use of spatial search strategies	Not significant

Our findings demonstrate that TREM2 deficiency accelerated neurodegeneration caused by TBI in hTau mice. Silencing of TREM2 in wild-type mice facilitates the hyperphosphorylation of endogenous tau, kinase activation, and neuroinflammation (23), although no neurodegenerative changes nor spatial learning deficits were observed. Similarly, TREM2 deficiency promotes tau pathology and aggregation *via* enhanced kinase activation in the mouse model of tauopathy (23, 40, 41). It has also been demonstrated that elevated levels of microglial-derived IL-1β induce tau pathology and neurodegeneration (42, 43). Furthermore, the chronic phase of hTau TBI mice showed persistent macrophage response that results in compromised spatial recognition (26). Thus, TBI-induced inflammation may be sufficient to promote tau pathology in hTau;*Trem2^-/-^* mice.

In contrast, opposing roles of TREM2 on tauopathy models have been reported. TREM2 deficiency or R47H variant of TREM2 in PS19 mice mitigates inflammatory gene expression and protects against brain atrophy at nine months (44, 45). This microglial opposing response might have resulted from the different genotypes or the stage of the disease examined. PS19 mice, which express a human tau transgene containing a P301S mutation, induce severe tangle deposition, gliosis, and neurodegeneration by nine months. Whereas hTau mice we used in our study, which express only human tau isoforms, develop mild age-associated tau pathology as indicated by accumulation of hyperphosphorylated tau begins from six months, and aggregated tau and paired helical filaments detectable at nine months. In 9 months PS19 model, microglia react strongly, and TREM2 may work detrimental. On the other hand, TREM2 may be beneficial in six months hTau model. Considering TREM2 also has different roles in AD-related myeloid cell functions early and late in disease progression (31, 46), TREM2 is generally protective for microglial activation and tau pathology in the early stage, then changes their phenotype to accelerate neurodegeneration and inflammation in a later stage.

Regional variations in TREM2 expression may contribute to the varying microglial function in the white matter. TREM2 deficiency hindered the accumulation of microglia with abnormal morphology with age, especially in the corpus callosum, which is rich in myelin that may trigger solid TREM2 signaling (18). In the cuprizone model of demyelination, TREM2 deficiency hampered the microglial response for myelin debris clearance, which led to axonal dystrophy and persistent demyelination. Other groups also showed that inhibiting microglial TREM2 expression impaired phagocytosis of apoptotic neurons (33) or the internalization of amyloid (31). In our hTau TBI model of neuroinflammation, mice were at six months of age in the chronic phase, where the microglial number does not differ between genotypes. Our data demonstrated that reactive microglia/macrophages were localized in the white matter where tau aggregation was enhanced in hTau;*Trem2^-/-^* TBI mice. Although co-labeling of phosphorylated tau with microglia/macrophage-specific Iba-1 was not performed, impaired phagocytosis of dystrophic neurons suggests a deficiency in clearance or phagocytosis of abnormal tau in this region (**Figure 6**). Tau pathology was much milder in hTau;*Trem2^-/-^* sham mice, which indicates TBI could further affect tau clearance. A previous study showed that TREM2 deficiency in hTau mice increased activation of the stress signaling JNK, GSKβ, ERK, and that downstream tau hyperphosphorylation and aggregation (40), supporting the idea that TREM2 deficiency competes with efficient tau clearance in the white matter.

Recently microglial transcriptomic states, termed disease-associated microglia (DAM) (47) and white matter-associated microglia (WAM) (48), have been identified. DAM is proposed to detect damage in the CNS partly through the TREM2 signaling pathway, and acquiring the DAM transcriptome requires a sequential two-stage process (47, 49). Because the transition from stage 1 (intermediate state) to stage 2 DAM (a full DAM signature) requires a TREM2 signal, microglia from TREM2 deficient mice that we used in this study might remain in an intermediate state. TBI appears to induce DAM2 signatures, which may serve neuroprotective functions and prevent tau pathology (50), whereas a loss of TREM2 function prevents microglia from switching to a full DAM2 state (47). These impaired TREM2 functions possibly prevent microglia from boosting phagocytosis, chemotaxis, and lipid metabolism and drive tau accumulation and neurodegeneration in hTau;*Trem2^-/-^* TBI mice. WAM, which shares parts of the DAM gene signature and depends on TREM2 signaling, might also be incompletely developed in TREM2-deficient mice.

BBB disruption has often been observed in patients with TBI (51). Microglia migrate to vessels in a CCR5-dependent manner to maintain BBB integrity after systemic inflammation (52). However, when inflammation persists, activated microglia impede the maintenance of tight junctions by releasing inflammatory modulators and phagocytosing astrocytic end-feet and endothelial cells, leading to impairment of BBB integrity and function (52–55). We observed abnormal BBB leakage in the white matter deep to the injured cortex of hTau;*Trem2^-/-^* mice, while little or no leakage was present in hTau TBI mice. It is plausible that TREM2-positive cells contribute to BBB restoration through unknown mechanisms. Since low TREM2-expressing microglia results in incomplete BBB (56), there is a possibility that hTau;*Trem2^-/-^* mice could have BBB vulnerability congenitally. This possibility has also been supported by other work showing impaired angiogenesis in TREM2-deficient mice in the ischemic stroke model (57). In addition, inflammatory mediators such as IL-6 caused by TBI may prevent BBB restoration in hTau;*Trem2^-/-^* TBI mice.

These results suggest that TREM2 deficiency amplifies chronic inflammation and facilitates neurodegeneration in the injured brain in hTau mice. Specifically, abnormal BBB leakage in the white matter highlights the crucial role of TREM2-positive cells in preventing the infiltration of peripheral macrophages. Further elucidation of TREM2 signaling is needed the clarify mechanisms underlying our observations and may provide deeper insights into the role of TREM2 in regulating inflammation following TBI. Future studies aim to seek the mechanisms between MAPT pathologies and TREM2 deficiency.

## Data availability statement

The original contributions presented in the study are included in the article/**Supplementary Material**. Further inquiries can be directed to the corresponding author.

## Ethics statement

The animal study was reviewed and approved by the Institutional Animal Care and Use Committee of the Cleveland Clinic.

## Author contributions

AK performed immunohistochemistry and wrote the manuscript. O.K-C. designed, performed surgeries and TBIs, and co-edited the manuscript. SB performed silver staining. GX helped with mouse strain maintenance. RR provided funding and expertise in data analysis and interpretation and co-edited the manuscript, and BL provided funding and expertise in data analysis and co-edited the manuscript. All authors contributed to the article and approved the submitted version.

## Funding

This work was supported by the Department of Defense grant (W81XWH-14-0265).

## Acknowledgments

We thank the Rodent Behavioral Core at the Lerner Research Institute within the Cleveland Clinic for support. We thank Dr. Imad Najm for the use of the fluid percussion device. We have permission to cite the conference paper of the World Congress of Neurology 2017 from Elsevier and Copyright Clearance Center (License number 5343890984223).

## Conflict of interest

The authors declare that the research was conducted in the absence of any commercial or financial relationships that could be construed as a potential conflict of interest.

## Publisher’s note

All claims expressed in this article are solely those of the authors and do not necessarily represent those of their affiliated organizations, or those of the publisher, the editors and the reviewers. Any product that may be evaluated in this article, or claim that may be made by its manufacturer, is not guaranteed or endorsed by the publisher.

## Abbreviations

AD, Alzheimer’s disease; FJC, fluoroJade C; MAPT, microtubule-associated protein tau; TBI, traumatic brain injury; TREM2, triggering receptor expressed on myeloid cells 2.
