# Supplementary material for: Triggering receptor expressed on myeloid cells 2 deficiency exacerbates injury-induced inflammation in a mouse model of tauopathy
Source: Front Immunol. 2022 Nov 1;13:978423. doi: 10.3389/fimmu.2022.978423 (PMC9664165; doi:10.3389/fimmu.2022.978423)
Supplement: Supplementary file 1 [file DataSheet_1.pdf]

Supplementary Figures

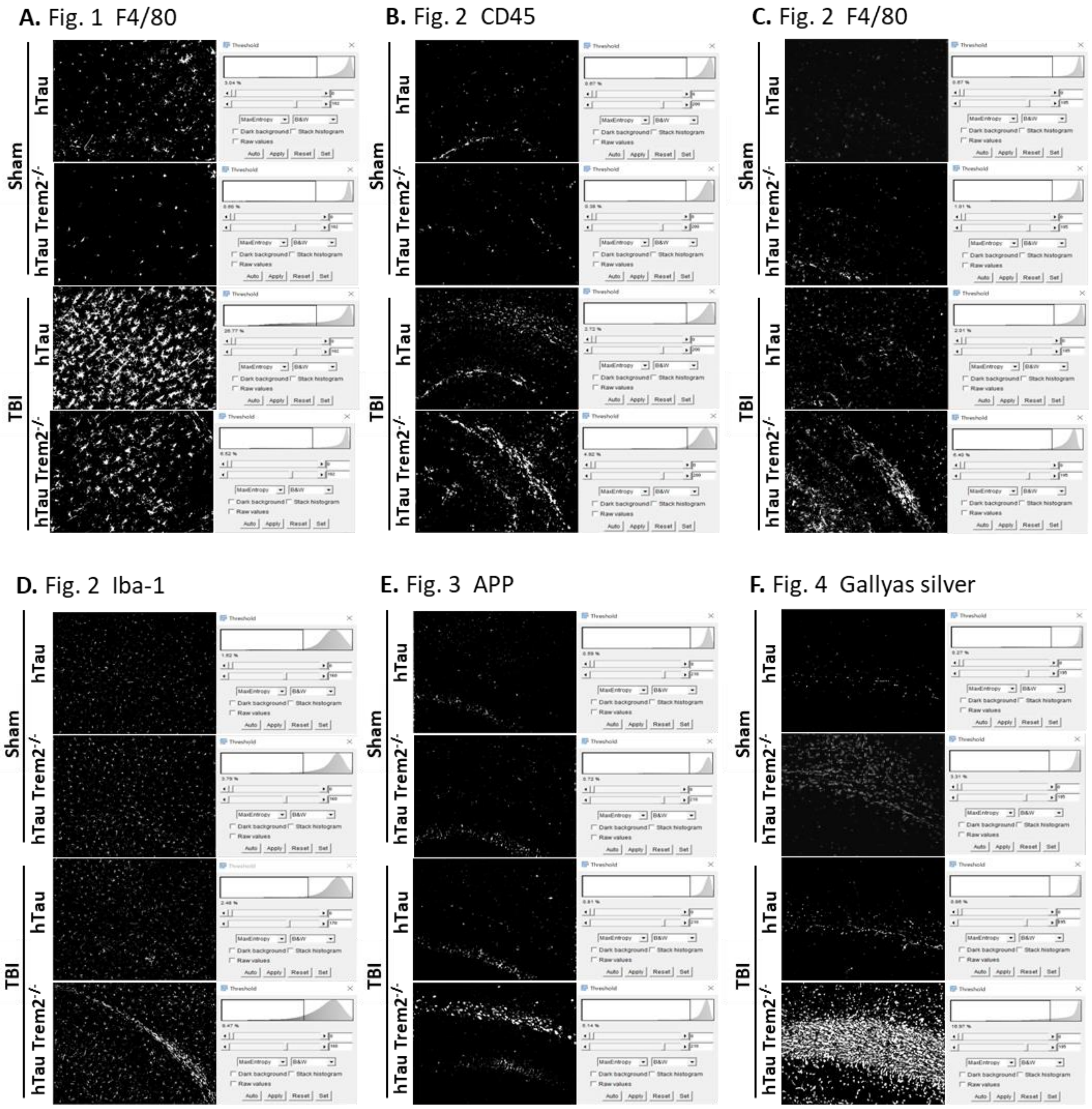

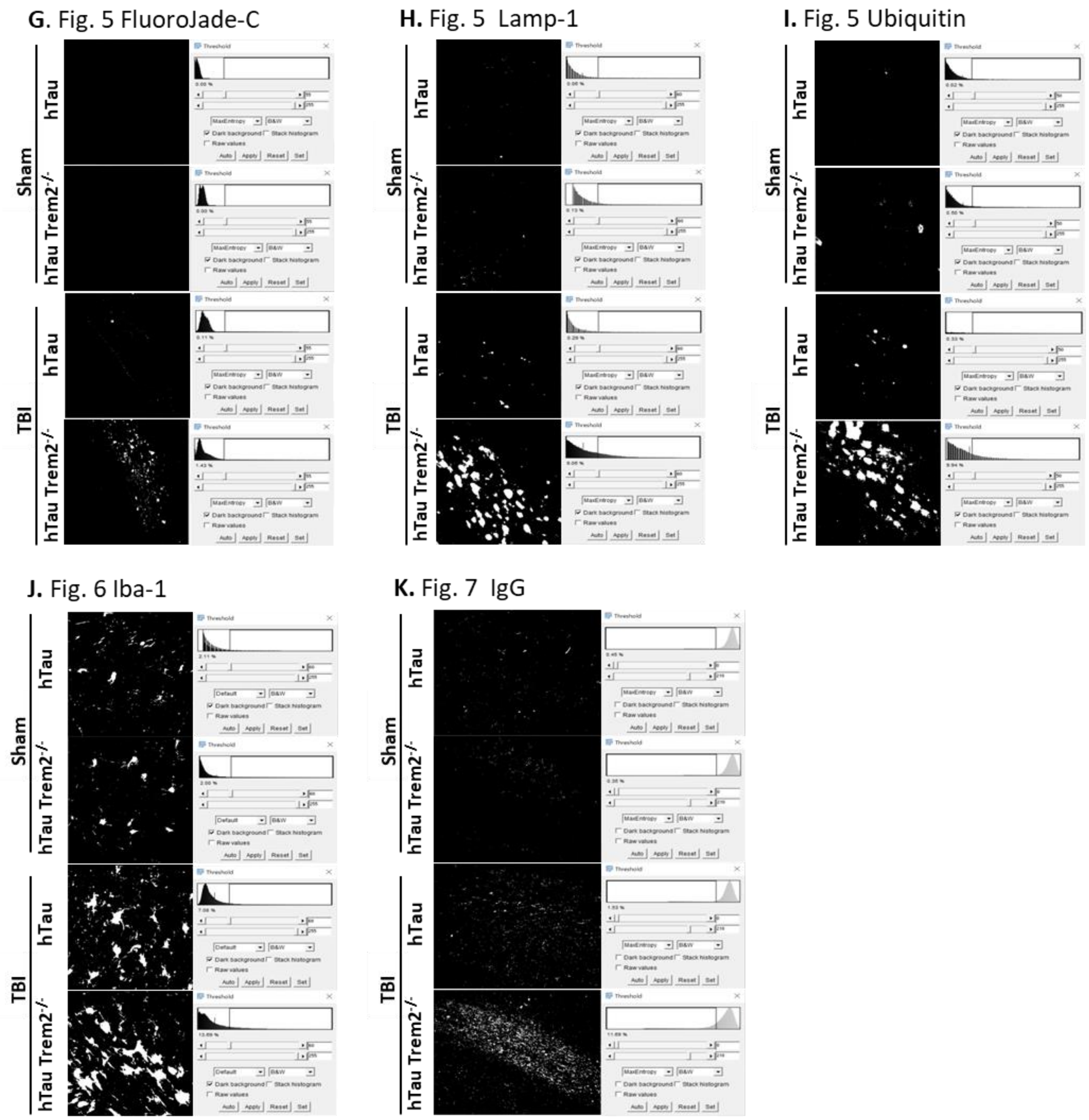

Supplementary Figure 1.

(A-K) The threshold used for the quantification of each immunoreactivity. Representative images of the pixel intensity histograms are shown.

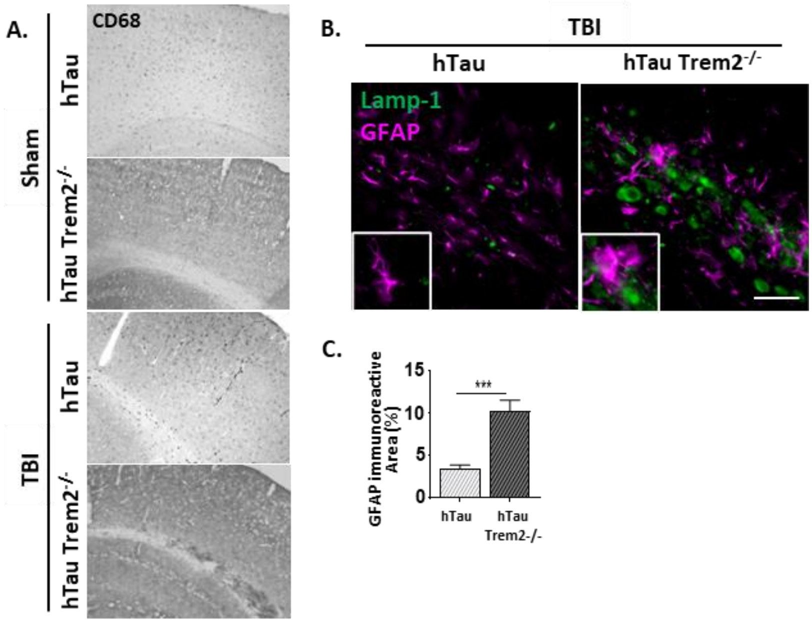

Supplementary Figure 2.

Htau;*Trem2*$^{-/-}$ TBI mice showed enhanced Iba1, CD45, and F4/80 in the white matter tract near the lesion at 120 DPI. Representative CD68 (A) and GFAP (B) staining intensity in TBI of hTau and hTau*Trem2*$^{-/-}$ mice in the white matter tract near lesion site. (C) Quantification of percent area covered by GFAP. n=5 mice per group. Error bars indicate SEM; ***p<0.0005. Scale bar 50μm.

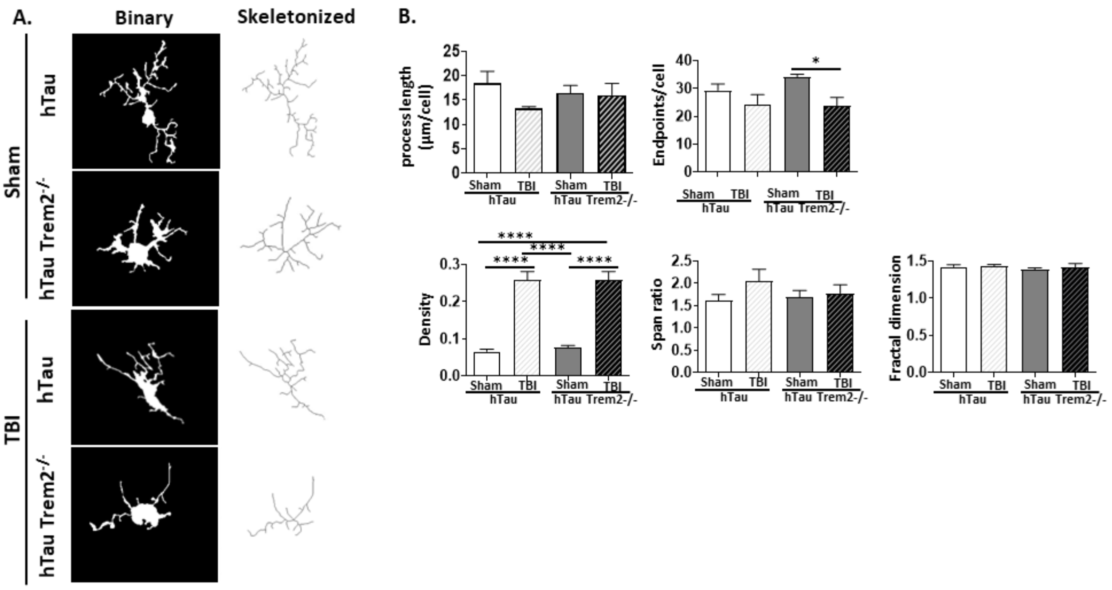

Supplementary Figure 3.

Microglia/macrophage morphological changes in hTau TBI mice and hTau;*Trem2*$^{-/-}$ TBI mice. (A) Representative binary and skeletonized images of microglia/macrophages in the white matter tract near the lesion from each group. Randomly selected Iba-1+ cells (20/group) were isolated, thresholded, and skeletonized. (B) Quantitative analysis of ramification (process length and endpoint), cell size (density), cell shape (span ratio), and cell complexity (fractal dimension). n=6 mice per group. Error bars indicate SEM; $*p < 0.05$, $****p < 0.0001$.
